# Up-regulated PIF1 predicts poor clinical outcomes and correlates with low immune infiltrates in clear cell renal cell carcinoma

**DOI:** 10.3389/fgene.2022.1058040

**Published:** 2023-01-04

**Authors:** Tong Cai, Ning Wang, Peng Meng, Weigui Sun, Yuanshan Cui

**Affiliations:** ^1^ Department of Urology, The Affiliated Hospital of Yangzhou University, Yangzhou University, Yangzhou, Jiangsu, China; ^2^ Department of Urology, The Affiliated YantaiYuhuangding Hospital of Qingdao University, Yantai, Shandong, China; ^3^ Department of Oncology, Yantai Traditional Chinese Medicine Hospital, Yantai, Shandong, China

**Keywords:** Pif1, clear cell renal cell carcinoma (ccRCC), immune, prognosis, biomarker

## Abstract

**Background:** Petite Integration Factor 1 (PIF1) is a multifunctional helicase and DNA processing enzyme that plays an important role in the process of several cancer types. However, the relationship between clear cell renal cell carcinoma (ccRCC) and PIF1 remains unclear. This study aims to explore the role of PIF1 in ccRCC tumorigenesis and prognosis.

**Methods:** Based on The Cancer Genome Atlas (TCGA) and the Gene Expression Omnibus (GEO) database, we retrieved and verified the expression of PIF1 in ccRCC tissues as well as normal tissues. To assess the protein expression of PIF1 by using the Human Protein Atlas and the Clinical Proteomic Tumor Analysis Consortium (CPTAC). We also performed receiver operating characteristic (ROC) curve analysis to differentiate the effectiveness of PIF1 in ccRCC and adjacent normal tissues. To evaluate the value of PIF1 on clinical outcomes in ccRCC patients by using multivariate methods and Kaplan‒Meier survival curves. Protein‒protein interaction (PPI) networks were made with STRING. We determined the relationship between the expression of PIF1 and immune cell infiltration with single-sample gene set enrichment analysis (ssGSEA).

**Results:** Compared with normal tissues, the expression of PIF1 was significantly elevated in ccRCC. The mRNA expression of PIF1 is correlated with high TNM stage and high pathologic stage. The receiver operating characteristic (ROC) curve analysis showed that PIF1 was related to an area under the curve (AUC) value of 0.928 to distinguish between ccRCC tissues and normal tissues. Kaplan‒Meier survival analysis showed that the overall survival (OS) of ccRCC patients with a high level of PIF1 was significantly shorter than that of those with a low level of PIF1. PIF1 may play an important role in the occurrence of tumors. Correlation analysis showed that PIF1-mediated carcinogenesis may participate in the process of tumor immune escape in ccRCC.

**Conclusion:** PIF1 could be a reference biomarker to identify ccRCC patients with poor prognosis. PIF1 may play a distinct role in the microenvironment of ccRCC by regulating tumor infiltration of immune cells, which is a new therapeutic target to affect the growth of the tumor.

## Introduction

Renal cell carcinoma (RCC) is one of the most common tumors in the urinary system, with approximately 300,000 new cases all over the world every year. (Ferlay et al., 2015). Patients who have renal cell carcinoma are between the ages of 60 and 70 years, which affects males twice as often as it affects females. (Lipworth et al., 2006). RCC is mainly divided into three histological subtypes: clear cell RCC (ccRCC), papillary RCC (pRCC) and chromophobe RCC (chRCC). The most common type of RCC is ccRCC, accounting for 75 percent of RCC cases. The least common type of RCC is chRCC (5%). (Ricketts et al., 2018). A great deal of epidemiological research has expressed that the risk factors associated with ccRCC are as follows: smoking, hypertension, overweight, regular exposure to chemical substances and others. (Lipworth et al., 2006). Currently, surgery is the main method for ccRCC treatment. Partial nephrectomy is a good treatment for localized RCC (T1a stage), which can protect renal function and have a satisfying prognosis; radical nephrectomy is adequate for patients with ccRCC in the T1b-T4 stages. (Ljungberg et al., 2015; Lucca et al., 2015). Surgery is not the best effective treatment for patients with advanced renal carcinoma or distant metastasis. Advanced renal carcinoma or patients with distant metastasis are treated mainly utilizing targeted therapy, immunotherapy, radiation therapy, etc. Different therapies have their advantages and disadvantages. Radiation therapy, which is a palliative treatment, has limited effects on ccRCC because ccRCC cells are not sensitive to radiation. (De Meerleer et al., 2014). Targeted therapy is an effective and safe treatment, and it mainly includes the following aspects: tyrosine kinase inhibitors, monoclonal antibodies against vascular endothelial growth factor (VEGF) bevacizumab, mammalian targets of rapamycin (mTOR) inhibitors and others, but the body will be tolerant to the medication by using them for a long time. (Bedke et al., 2017; Choueiri and Motzer, 2017). Immunotherapy is a new kind of method to treat ccRCC; however, it can be easily affected by many factors of ccRCC, including the expression of neoantigens, the microbiome, and genetics. (Chen and Mellman, 2017). Overall, the development of ccRCC is a multifactor and multistage process, and the choice of treatment can be affected by a variety of factors. Therefore, it is inevitable to deeply understand the pathogenesis and molecular mechanism of ccRCC, which may provide a direct precision treatment and new treatment strategy for ccRCC.

Petite Integration Factor 1 (PIF1) Pif1 is a multifunctional helicase and DNA processing enzyme that plays a critical role in maintaining genomic stability in higher animals and plants. (Dehghani-Tafti et al., 2019). According to whether the first or second AUG is used to start translation, there are two forms of Pif1: one is stored in mitochondria, and the other is localized to nuclei. (Belmonte et al., 2019). Pif1 is a protein-coding gene. The gene encodes a DNA-dependent adenosine triphosphate (ATP) metabolic enzyme, which functions as a 5 ′to 3′ DNA helicase. (Wu and Hickson, 2006; Galletto and Tomko, 2013; Pohl and Zakian, 2019). It effectively unwinds the G-quadruplex (G4) DNA structure and forks RNA‒DNA hybrids. (Deegan et al., 2019). Resolves the G4 structure, preventing replication pausing and double-strand breaks (DSBs) at G4 motifs. Meanwhile, it also participates in the maintenance of telomeric DNA. Inhibits telomere elongation, *de novo* telomere formation and telomere addition to DSBs *via* catalytic inhibition of telomerase. (Phillips et al., 2015). Research has found that Pif1 can remove telomerase from the end of DNA, and it did not affect the binding levels of telomere constitutive protein both *in vitro* and certainly *in vivo*. (Boulé et al., 2005). The cellular function of PiF1 is not known at present. PIF1 may help to maintain replication fork progression in tumors, especially during replication stress induced by genotoxic drugs. (Gagou et al., 2011; Gagou et al., 2014). Therefore, some scholars have suggested that PIF1 is an important target in cancer treatment. However, the relationship between ccRCC and PIF1 is still unclear.

In this study, we collected valuable reference data and carried out data analyses from different databases (TCGA, GEO and Human Protein Atlas). We also evaluated a link between PIF1 expression, clinical data and overall survival (OS) in patients with ccRCC. Then, we determined the relationship between PIF1 expression and immune-related genes and proteins by using the TIMER and GEPIA databases. At the end of this study, we also studied the relevant information on the PIF1-interacting protein network through the STRING website. Finally, we found that PIF1 may be useful for early diagnosis and predicting the prognosis of ccRCC. This research provides a new idea for the potential mechanism of ccRCC tumorigenesis, and PIF1 can be used as a potential biomarker for the diagnosis and prognosis of ccRCC.

## Methods and materials

### The Cancer Genome Atlas and the Gene Expression Omnibus

The RNA-Seq expression and corresponding pathology clinical data of ccRCC patients were obtained from the official TCGA website (https://genome-cancer.ucsc.edu/). The Cancer Genome Atlas (TCGA) database is an open large-scale platform distributed free of cancer genomes, and it contains the pathological and clinical data of more than 30 different types of cancer. The Gene Expression Omnibus (GEO) database is a public functional genomics dataset (https://www.ncbi.nlmnih.gov/geo/) that can help users query and download curated gene expression profiles for free. All data for this study were obtained through open access from the TCGA and GEO databases, and the research did not require approval from the local ethics committee.

### The Human Protein Atlas , UALCAN, and Clinical Proteomic Tumor Analysis Consortium

The HPA was divided into three large chapters: Cell, Tissue and Pathology. Protein expression in human normal tissues, tumor tissues, and cells. This database was made up of information on cell-specific locations of more than 40 different healthy tissues and more than 20 different types of cancer. Meanwhile, this research used HPA to compare the protein expression of PIF1 between normal tissue and ccRCC tissues. In addition, we can also obtain data about protein immunohistochemistry in human tumor tissues and normal tissues from the HPA website. The UALCAN database (http://ualcan.path.uab.edu/) can be used to analyse differential gene expression in cancer and normal tissues online. This study analysed the relationship between PIF1 gene expression and clinical features by using the UALCAN database. CPTAC (http://ualcan.path.uab.edu/analysis-prot.html) analyses tumor biospecimens by using mass spectrometry with the application of proteomic technologies, identifying and quantifying the constituent proteins and characterizing the proteins of every tumor sample. In this study, we analysed the PIF1 protein expression obtained from CPTAC by using the UALCAN database.

### Univariate and multivariate logistic regression analysis

Univariate Cox regression was used to analyse the relationships between the OS of patients across two different queues and the expression level of PIF1, which helped define the impact of PIF1 expression in ccRCC patients. Then, we used multivariate analysis to evaluate whether PIF1 is an independent prognostic factor of survival for ccRCC patients. When *p* < 0.05, it indicated that there was statistically significant PIF1 in the Cox regression analysis.

### Protein‒protein interaction network and functional enrichment analysis

The Search Tool for the Retrieval of Interacting Genes/Proteins (STRING) website (https://string-db.org/) is another online database that can be used to construct PPI networks and screen hub genes. We obtained PPI network information after importing PIF1 into the STRING database. When the confidence score was >0.7, it indicated that there was a statistically significant difference. Gene Ontology (GO) enrichment and Kyoto Encyclopedia of Genes as well as Genomes (KEGG) pathway analyses of coexpressed genes were analysed by using the “ClusterProfiler” package and visualized by the “ggplot2” package. (Wickham et al., 2016).

### Gene set enrichment analysis

In the present study, the single-sample GSEA method from the R package “GSV A″ was used to show the infiltration enrichment of 24 common immune cells, including T helper (Th) cells, cytotoxic cells, CD8^+^ T-cell, T-cell, T effector memory (Tem), NK CD56bright cells, regulatory T-cell (Treg), type 1 Th cells (Th1), activated DCs (aDCs), T follicular helper (Tfh), Tcentral memory (Tcm), plasmacytoid DCs (pDCs), NK CD56dim cells, type 2 Th cells (Th2), B-cell, dendritic cells (DCs), natural killer (NK) cells, macrophages, immature DCs (iDCs), type 17 Th cells (Th17), eosinophils, neutrophils, mast cells, and T gamma delta (Tgd). Then, we used Spearman’s analysis to evaluate the relationship between immune cell infiltration and PIF1 expression. We also compared the infiltration levels of immune cells between the high-PIF1 expression group and the low-PIF1 expression group by using the Wilcoxon rank-sum test.

### Gene expression profiling interactive analysis

The GEPIA (http://gepia.cancer-pku.cn/index.html) database includes a total of 8,587 normal samples and 9,736 tumor samples from TCGA and GTEx data, and we can obtain the corresponding data online. The database analyses the expression of RNA sequencing in different types of samples. This study used it to analyse the relationship between PIF1 expression and various immune cell markers, with the *x*-axis representing the amount of PIF1 expression and the *y*-axis representing other types of genes of interest. In addition, this study used TIMER data to identify which genes had a significant correlation with PIF1 expression, as shown by the GEPIA website.

#### Tumor-immune system interaction database

The TISIDB database (http://cis.Hku.hk/TISIDB/) integrates multiple heterogeneous data web portals, which can obtain relevant information about the tumors and the innate immune system. This study establishes the expression of PIF1 and tumour-infiltrating lymphocytes (TILs) in cancers occurring in humans by using this platform. According to the gene expression profile, we used gene set variation analysis to infer the relative abundance of TILs. The relationships between PIF1 and TILs were quantified by Spearman’s test.

### Statistical analyses

R (V 3.6.3)9 was utilized for all statistical analyses, and the R package ggplot2 was used to determine the expression differences. The Mann‒Whitney *U* test and paired *t*-test were used to establish the differences between ccRCC tissues and adjacent normal tissues. The ROC curve can detect the cut-off value of PIF1 by using the pROC package. Kaplan‒Meier and log-rank tests were performed to evaluate the effect of PIF1 on survival by using the survminer package. Correlation analysis was utilized by Pearson correlation and Spearman’s test.

## Result

### Expression pattern of PIF1 in a pan-cancer perspective

**FIGURE 1:**
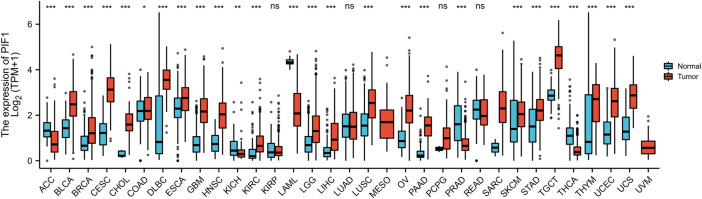
Expression pattern of PIF1 from a pancancer perspective. The mRNA expression of PIF1 was upregulated in 21 of 33 cancer types and downregulated in 5 of all 33 cancer types compared with normal tissues. (ns: *p* ≥ 0.05; *: *p* < 0.05; **: *p* < 0.01; ***: *p* < 0.001).

We evaluated the mRNA expression pattern of PIF1 in thirty-three types of cancer. As shown in Figure 1, the results from the database showed that PIF1 was significantly upregulated in 21 out of all 33 cancer types compared with normal tissues, such as testicular germ cell tumor (TGCT), bladder urothelial carcinoma (BLCA), cervical squamous cell carcinoma and endocervical adenocarcinoma (CESC). However, decreased PIF1 expression was found in 5 of 33 cancer types, including adrenocortical carcinoma (ACC), kidney chromophobe (KICH), prostate adenocarcinoma (PRAD) and others. As a result, the data suggest that PIF1 mRNA is abnormally expressed in various types of cancers.

### Upregulated mRNA and protein expression of PIF1 in ccRCC patients

**FIGURE 2:**
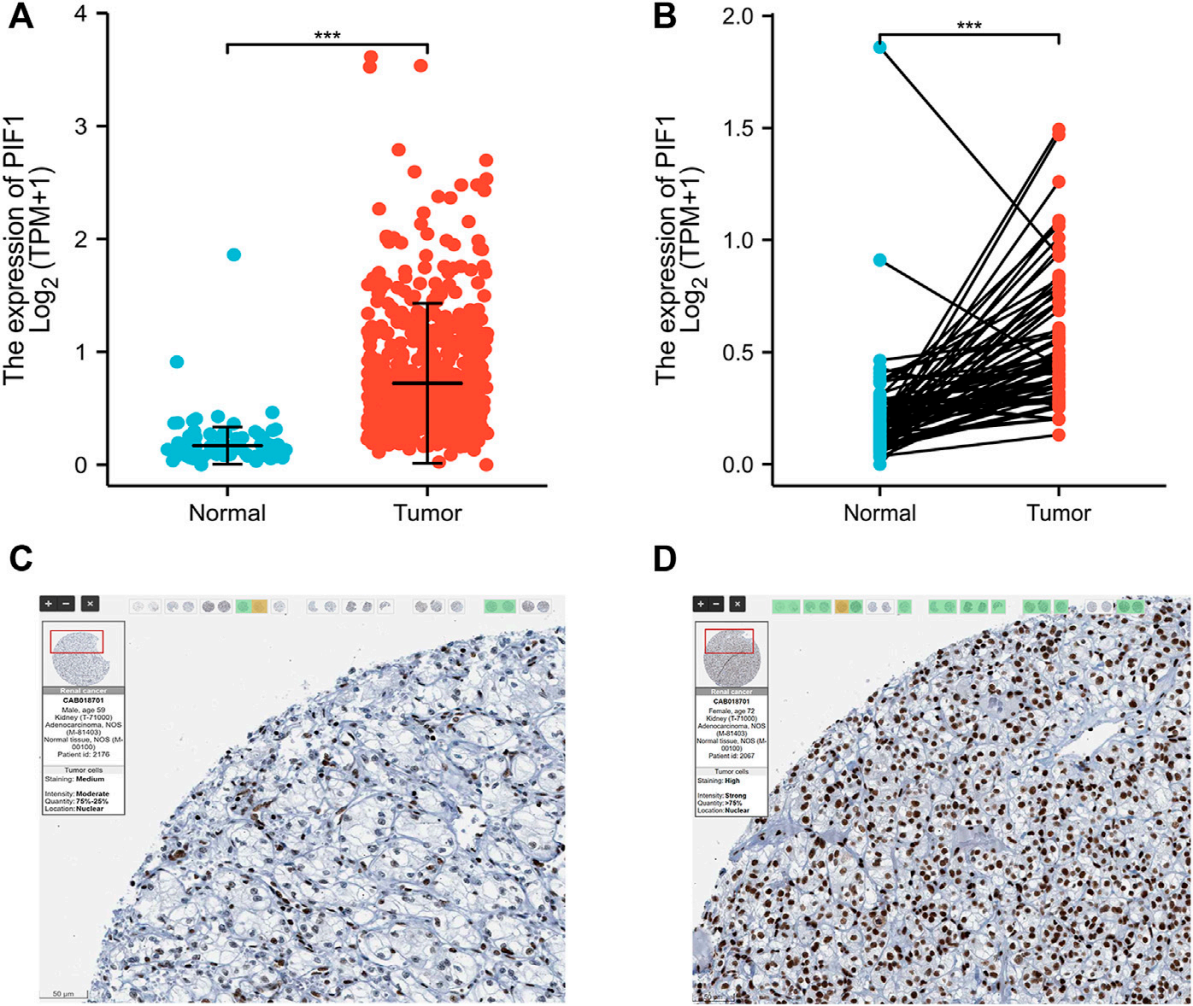
The mRNA and protein expression of PIF1 in ccRCC. **(A)** mRNA expression levels of PIF1 in 611 ccRCC samples and 72 normal samples. **(B)** mRNA expression levels of PIF1 in 72 ccRCC and matched adjacent normal samples. **(C)** Normal tissues: the protein levels of PIF1 based on the Human Protein Atlas. **(D)** Tumor tissues: the protein levels of PIF1 based on the Human Protein Atlas. (***p < 0.001). ccRCC: Clear Cell Renal Cell Carcinoma.

To determine the protein and mRNA expression of PIF1 in ccRCC, this study analysed PIF1 expression data from the TCGA, GEO and HPA databases. As shown in Figure 2A, compared with the adjacent normal tissues (*n* = 611), the unpaired data analysis showed that the mRNA expression levels of PIF1 in ccRCC increased significantly (*n* = 72) (0.83 ± 0.544 vs. 0.213 ± 0.239, *p* < 0.001). Figure 2B shows that the mRNA expression levels of PIF1 in ccRCC tissues (*n* = 72) were significantly higher than those in adjacent normal tissues (*n* = 72) (Figure 2B, 0.559 ± 0.297 vs. 0.213 ± 0.239, Mann‒Whitney *U* test, *p* < 0.001). The results of immunohistochemistry staining of HPA showed that PIF1 protein expression was also upregulated in ccRCC tissue, as shown in Figures 2C,D. These findings indicated that both the mRNA and protein expression of PIF1 were upregulated in ccRCC.

### Relationships between PIF1 mRNA levels and clinical pathological features of ccRCC patients

**FIGURE 3:**
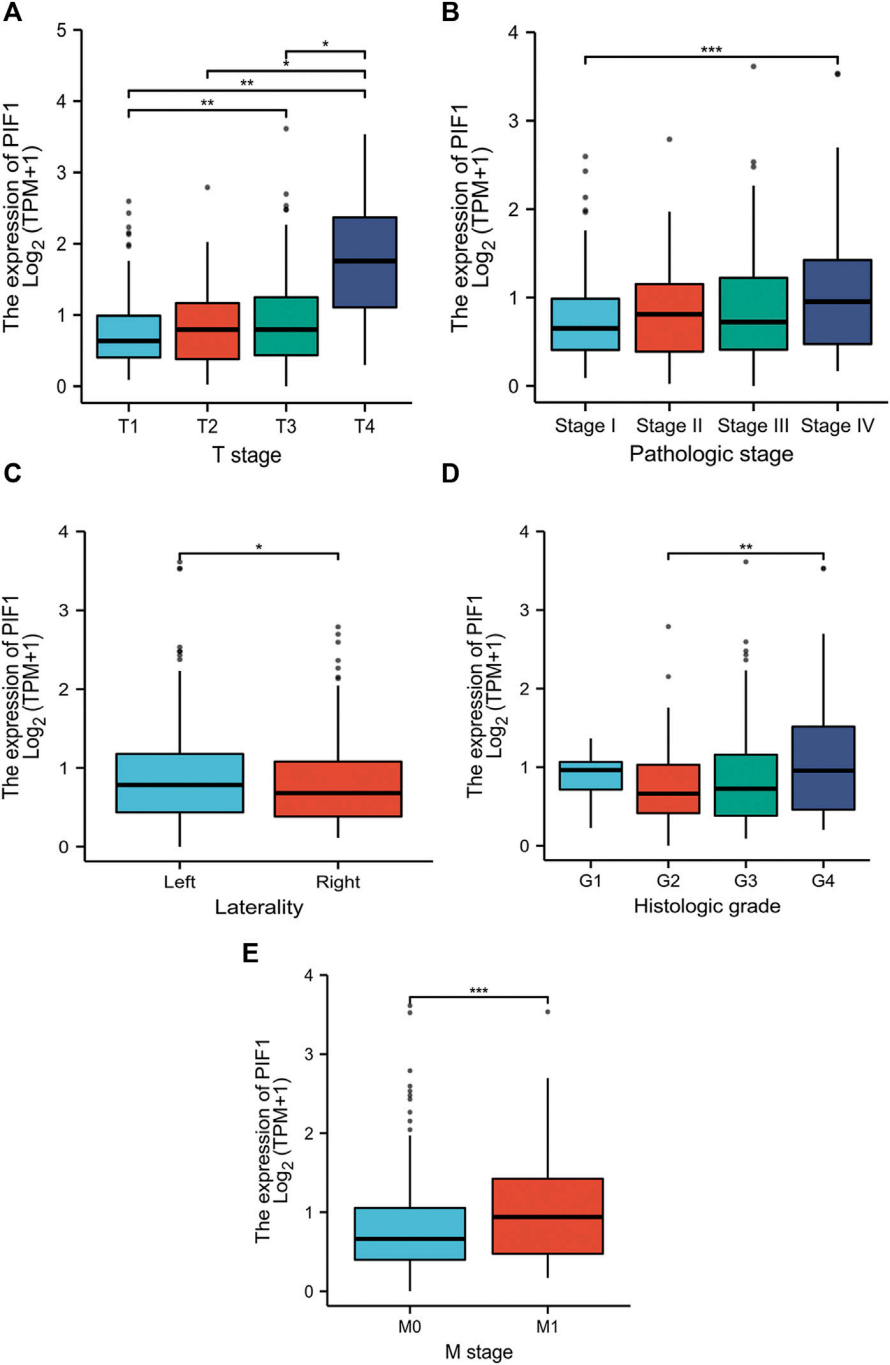
Relationships between PIF1 mRNA levels and clinical pathological characteristics. Higher expression levels of PIF1 were observed in patients with high T stage **(** A **)**, patients with high pathologic stage **(** B **)**, patients with left ccRCC **(** C **)**, patients with histologic grade **(** D **)**, and patients with M stage **(** E **)**. (ns, no significance, *p < 0.05, **p < 0.01, ***p < 0.001).

In this study, we performed Dunn’s test and the Kruskal‒Wallis test to evaluate the correlation between the mRNA expression of PIF1 and the clinicopathological characteristics of ccRCC samples. The baseline characteristics of ccRCC patients from the TCGA database are shown in Table 1. As shown in Figures 3A–E, higher expression levels of PIF1 were identified in ccRCC patients with high T stage (Figure 3A), patients with high pathologic stage (Figure 3B), patients with left ccRCC (Figure 3C), patients with histologic grade (Figure 3D), and patients with M stage (Figure 3E). Overall, these results indicate that PIF1 is correlated with high TNM stage and prove that PIF1 can be an important reason for poor prognosis in patients with ccRCC.

**TABLE 1:** The clinical characteristics of ccRCC patients between the high PIF1 expression group and low expression group.

Characteristic	Low expression of PIF1	High expression of PIF1	*p*
n	269	270	
Age, n (%)			0.763
≤60	132 (24.5%)	137 (25.4%)	
>60	137 (25.4%)	133 (24.7%)	
Gender, n (%)			1.000
Female	93 (17.3%)	93 (17.3%)	
Male	176 (32.7%)	177 (32.8%)	
T stage, n (%)			0.003
T1	158 (29.3%)	120 (22.3%)	
T2	31 (5.8%)	40 (7.4%)	
T3	78 (14.5%)	101 (18.7%)	
T4	2 (0.4%)	9 (1.7%)	
N stage, n (%)			0.279
N0	117 (45.5%)	124 (48.2%)	
N1	5 (1.9%)	11 (4.3%)	
M stage, n (%)			0.001
M0	235 (46.4%)	193 (38.1%)	
M1	27 (5.3%)	51 (10.1%)	
Pathologic stage, n (%)			0.002
Stage I	154 (28.7%)	118 (22%)	
Stage II	25 (4.7%)	34 (6.3%)	
Stage III	61 (11.4%)	62 (11.6%)	
Stage IV	28 (5.2%)	54 (10.1%)	
Histologic grade, n (%)			0.011
G1	4 (0.8%)	10 (1.9%)	
G2	130 (24.5%)	105 (19.8%)	
G3	103 (19.4%)	104 (19.6%)	
G4	27 (5.1%)	48 (9%)	

### Differential RNA-Seq levels of PIF1 as a potential biomarker to differentiate between ccRCC and normal samples

**FIGURE 4:**
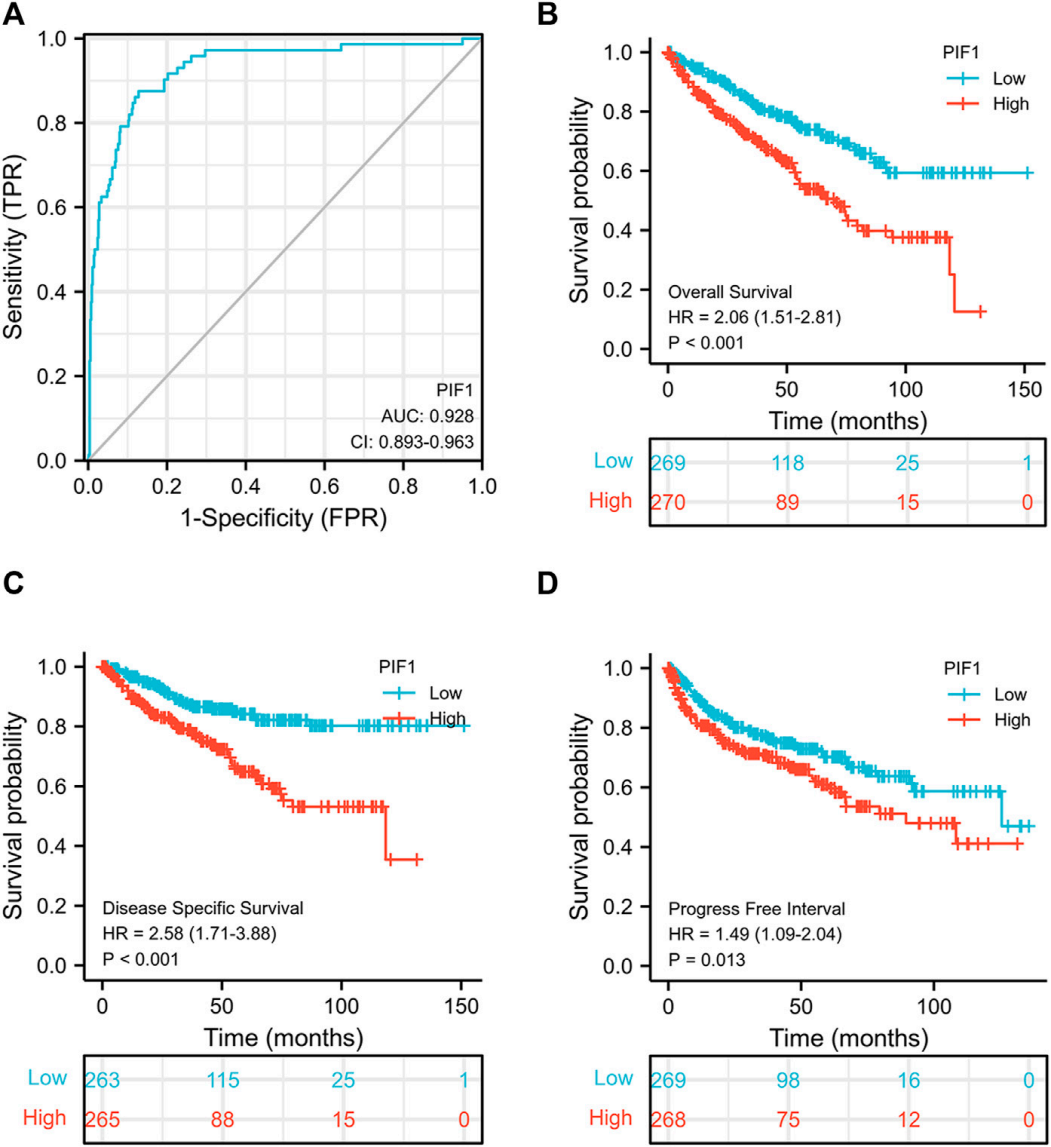
ROC and Kaplan‒Meier curves for PIF1. **(A)** ROC curve showed that PIF1 had an AUC value of 0.928 to discriminate ccRCC tissues from healthy controls. **(B–D)** Kaplan‒Meier survival curves indicated that ccRCC patients with high PIF1 mRNA expression had a shorter OS, DSS and PFI than those with low PIF1 mRNA expression. OS: overall survival; DSS: disease-specific disease; PFI: progression-free interval.

To study the value of PIF1 in distinguishing ccRCC samples from normal samples, ROC curve analysis was conducted. The ROC curve analysis showed that PIF1 was related to an AUC value of 0.928 (95% CI: 0.893–0.963), as shown in Figure 4A. Based on a cut-off value of 0.315, PIF1 showed a sensitivity, specificity, and accuracy of 87.5, 87.2, and 74.7%, respectively. As shown in Table 2, in univariate analysis, T stage (*p* < 0.001), N stage (*p* < 0.001), M stage (*p* < 0.001), pathologic stage (*p* < 0.001), histologic grade (*p* < 0.001), and PIF1 expression (*p* < 0.001) were related to overall survival (OS); in multivariate analysis, only M stage (*p* < 0.002), histologic grade (*p* = 0.036), and PIF1 expression (*p* = 0.002) could act as independent prognostic factors for ccRCC (Table 2). These findings imply that PIF1 could be used as a reference biomarker to effectively differentiate ccRCC from normal tissues.

**TABLE 2:** Univariate and multivariate Cox proportional hazards analyses of PIF1 expression and OS for patients with ccRCC.

Characteristics	Total (N)	Univariate analysis		Multivariate analysis	
		Hazard ratio (95% CI)	P value	Hazard ratio (95% CI)	P value
T stage	539				
T1&T2	349	Reference			
T3&T4	190	3.228 (2.382-4.374)	**<0.001**	1.259 (0.552-2.870)	0.584
N stage	257				
N0	241	Reference			
N1	16	3.453 (1.832-6.508)	**<0.001**	1.435 (0.715-2.880)	0.310
M stage	506				
M0	428	Reference			
M1	78	4.389 (3.212-5.999)	**<0.001**	2.342 (1.368-4.010)	**0.002**
Pathologic stage	536				
Stage I&Stage II	331	Reference			
Stage III&Stage IV	205	3.946 (2.872-5.423)	**<0.001**	1.572 (0.612-4.038)	0.347
Histologic grade	531				
G1&G2	249	Reference			
G3&G4	282	2.702 (1.918-3.807)	**<0.001**	1.709 (1.036-2.821)	**0.036**
Laterality	538				
Left	252	Reference			
Right	286	0.706 (0.523-0.952)	**0.023**	1.094 (0.710-1.687)	0.683
PIF1	539				
Low	269	Reference			
High	270	2.061 (1.511-2.812)	**<0.001**	2.046 (1.290-3.246)	**0.002**

ccRCC, Clear Cell Renal Cell Carcinoma; OS, overall survival.

### High mRNA expression of PIF1 is correlated with poor OS, disease-specific survival and progression-free interval

This study generated Kaplan‒Meier curves to explore the correlation between PIF1 mRNA expression and OS, DSS and PFI in ccRCC patients. As shown in Figure 4B, the OS of ccRCC patients with high PIF1 levels was significantly shorter than that of those with low PIF1 levels (hazard ratio (HR) = 2.06 (1.51–2.81), *p* < 0.001). Meanwhile, Figures 4C,D also show that the DSS and PFI in ccRCC patients with a high level of PIF1 were significantly shorter than those in ccRCC patients with a low level of PIF1 (hazard ratio (HR) = 2.58 (1.71–3.88), *p* < 0.001; HR = 1.49 (1.09–2.04), *p* = 0.013). In summary, the high mRNA expression of PIF1 could be used as a reference biomarker of worse prognosis in ccRCC.

### Construction and verification of a nomogram based on PIF1 expression

**FIGURE 5:**
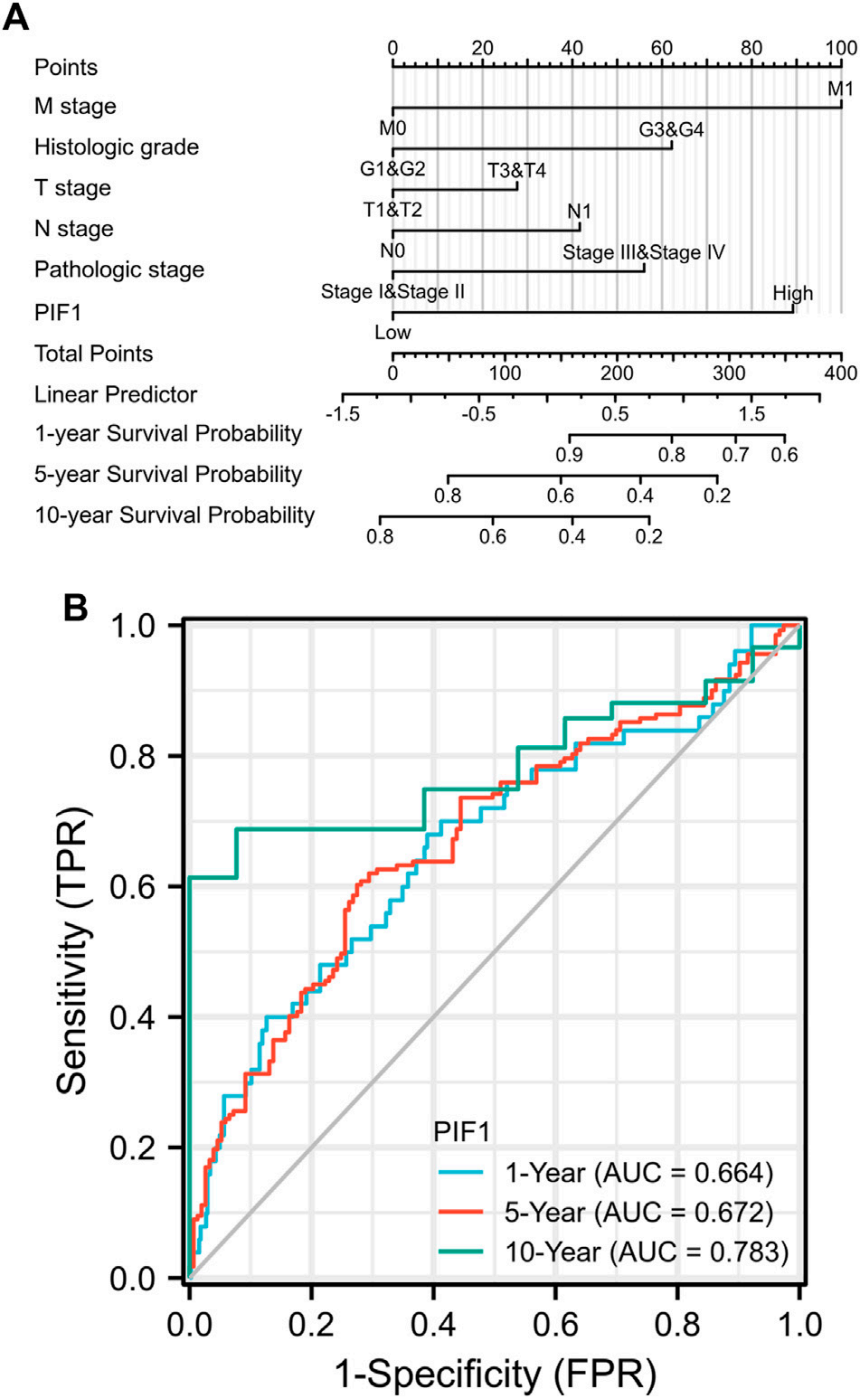
**(A)** Nomogram for predicting the probability of 1-, 5-, and 10-year OS for ccRCC patients. **(B)** The ROC curve analysis also showed that PIF1 was associated with AUC values of 0.664 (1 year), 0.672 (5 years), and 0.783 (10 years). ccRCC: clear cell renal cell carcinoma; OS: overall survival.

To help provide a useful quantitative model, we built a nomogram to help clinicians determine the correct prognosis of patients with ccRCC. The nomogram combined the clinical features of patients who were independently correlated with survival by using multivariate analysis (M stage, histologic grade and PIF1; Figure 5A). Based on the multivariate Cox analysis, we used a point scale to assign the locations of these variables in the nomogram, as shown in Figure 5B: we used a straight line to determine the number of points for the variables in the nomogram and rescaled the total number of the points appointed to each variable within the range of 0–100. Then, the different locations of the variables are summed and listed as the total number of points. Vertical lines were drawn from the axis of total points downwards to the outcome axis to determine the expected survival of ccRCC patients after 1, 5, and 10 years. As shown in Figure 5B, this result proved that the ROC curve analysis also showed that PIF1 was associated with AUC values of 0.664 (1 year), 0.672 (5 years), and 0.783 (10 years). To summarize, the results indicated that the nomogram and ROC curve analysis are excellent models capable of establishing long-term survival (1, 5, and 10 years) in ccRCC patients compared with individual prognostic factors.

### Identifying DEGs in the high and low PIF1 expression groups

**FIGURE 6:**
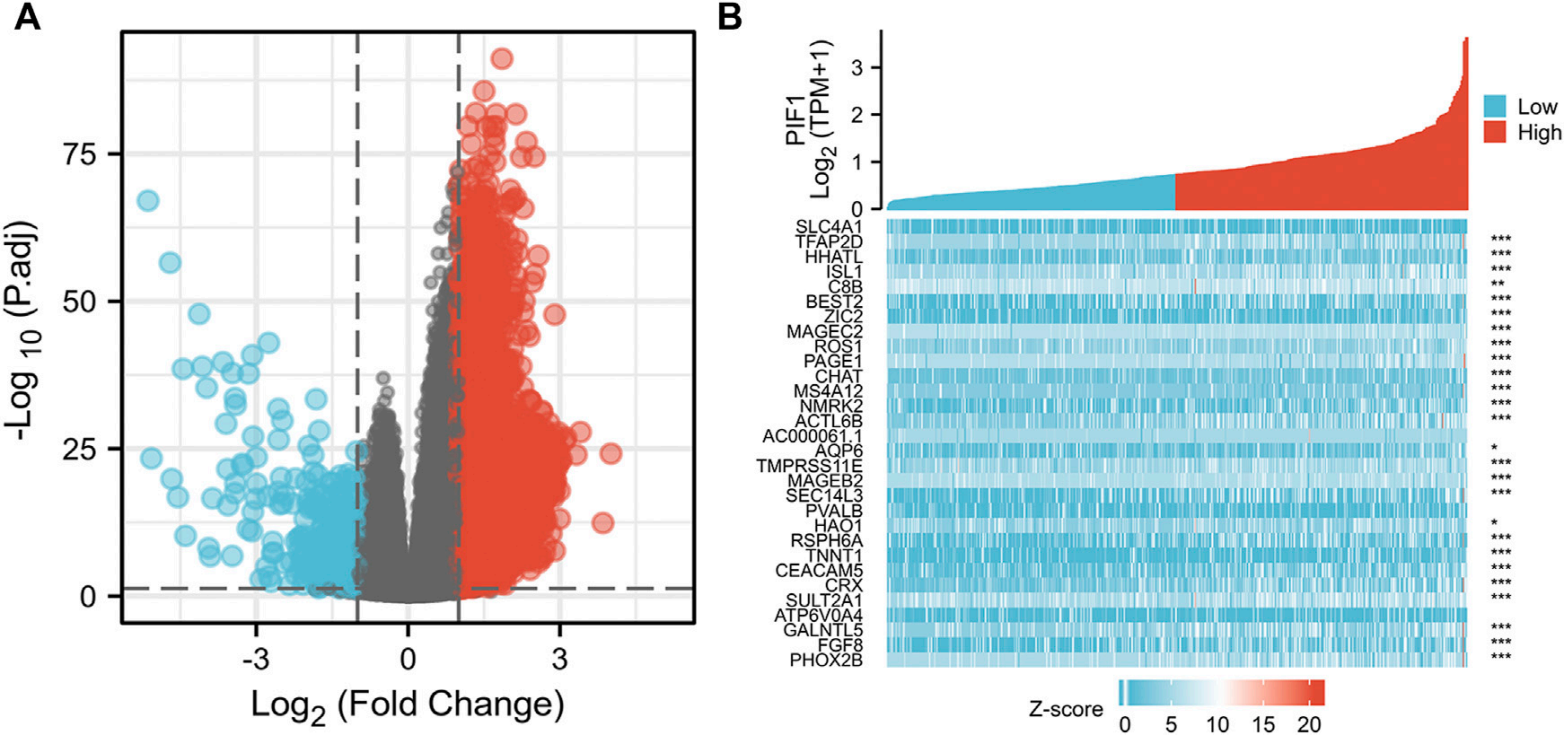
Differentially expressed genes between patients with high and low PIF1 expression. **(A)** Volcano plot of differentially expressed genes between the high and low PIF1 expression groups. Normalized expression levels are shown in descending order from green to red. **(B)** Heatmap of the top ten significantly differentially expressed genes between the high and low PIF1 expression groups. Green and red dots represent downregulated and upregulated genes, respectively.

We used the DSEeq2 package in R (|logFC|>2, modified *p*-value <0.05) to analyse the data from the TCGA database and found 1,331 DEGs in the high level expression of the PIF1 group and low level expression of the PIF1 group. Among them, 48 genes were upregulated and 133 were downregulated in the high-level expression group (Figure 6A). Figure 6B shows the heatmap of the ten most significant DEGs in the high-level and low-level PIF1 expression groups.

### PPI networks and functional annotations

**FIGURE 7:**
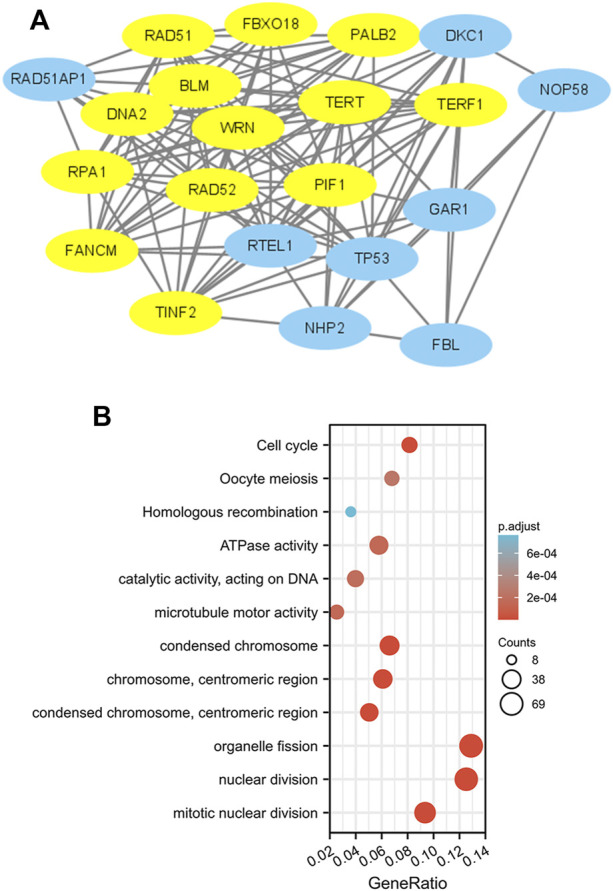
PPI networks and functional enrichment analyses. **(A)** A network of PIF1 and its coexpressed genes. **(B)** Functional enrichment analyses of 12 involved genes. PIF1 was associated with cytokine‒cytokine receptor interactions.

To construct PPI networks and functional annotations, the STRING database, GO, and KEGG analyses were performed. Figure 7A shows a network of PIF1 and its 21 related coexpressed genes. As shown in Figure 7B, the changes in the biological process of PIF1 were related to organelle fission, nuclear division and mitotic nuclear division. Functional annotations have shown that these types of genes might be associated with homologous recombination.

### Correlation analysis of PIF1 expression and immune cell infiltration in ccRCC

**FIGURE 8:**
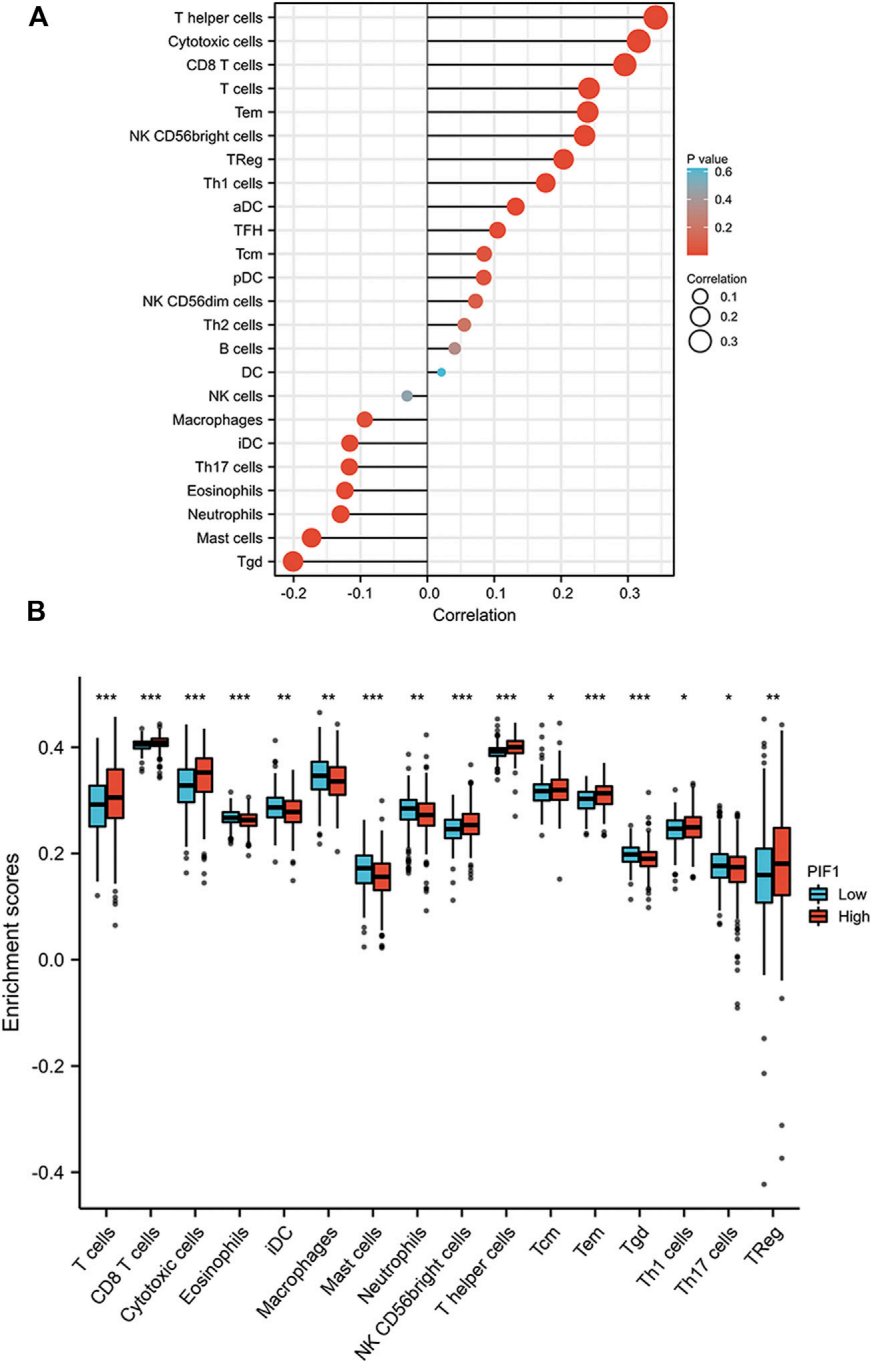
Correlations of PIF1 expression with immune infiltration level. **(A)** Relationships among the infiltration levels of 24 immune cell types and PIF1 expression profiles by Spearman’s analysis. **(B)** Comparison of the infiltration levels of the most correlated immune cells, including T-cell, CD8^+^ T-cell, cytotoxic cells, eosinophils, immature DCs (iDCs), macrophages, mast cells, neutrophils, NK CD56dim cells, T helper (Th) cells, Tcentral memory (Tcm), T effector memory (Tem), T gamma delta (Tgd), type 1 Th cells (Th1), type 17 Th cells (Th17), and regulatory T-cell (Treg).

First, we used the ssGSEA method to determine the infiltration of 24 immune cell types in ccRCC. Then, we also researched the association between PIF1 and immune cell infiltration by using Spearman’s analysis. As Figure 8A shows, the expression of PIF1 was positively correlated with the expression of T helper (Th) cells, cytotoxic cells, T-cell and CD8^+^ T-cell. However, T gamma delta (Tgd), mast cells, neutrophils and immature DCs (iDCs) were negatively correlated with PIF1. In particular, we also assessed the infiltration levels of sixteen relevant immune cells in distinct PIF1 groups, and the results coincided with the results shown in Figure 8B.

### Relationship between PIF1 and PD1/PD-L1/CTLA4 in ccRCC

**FIGURE 9:**
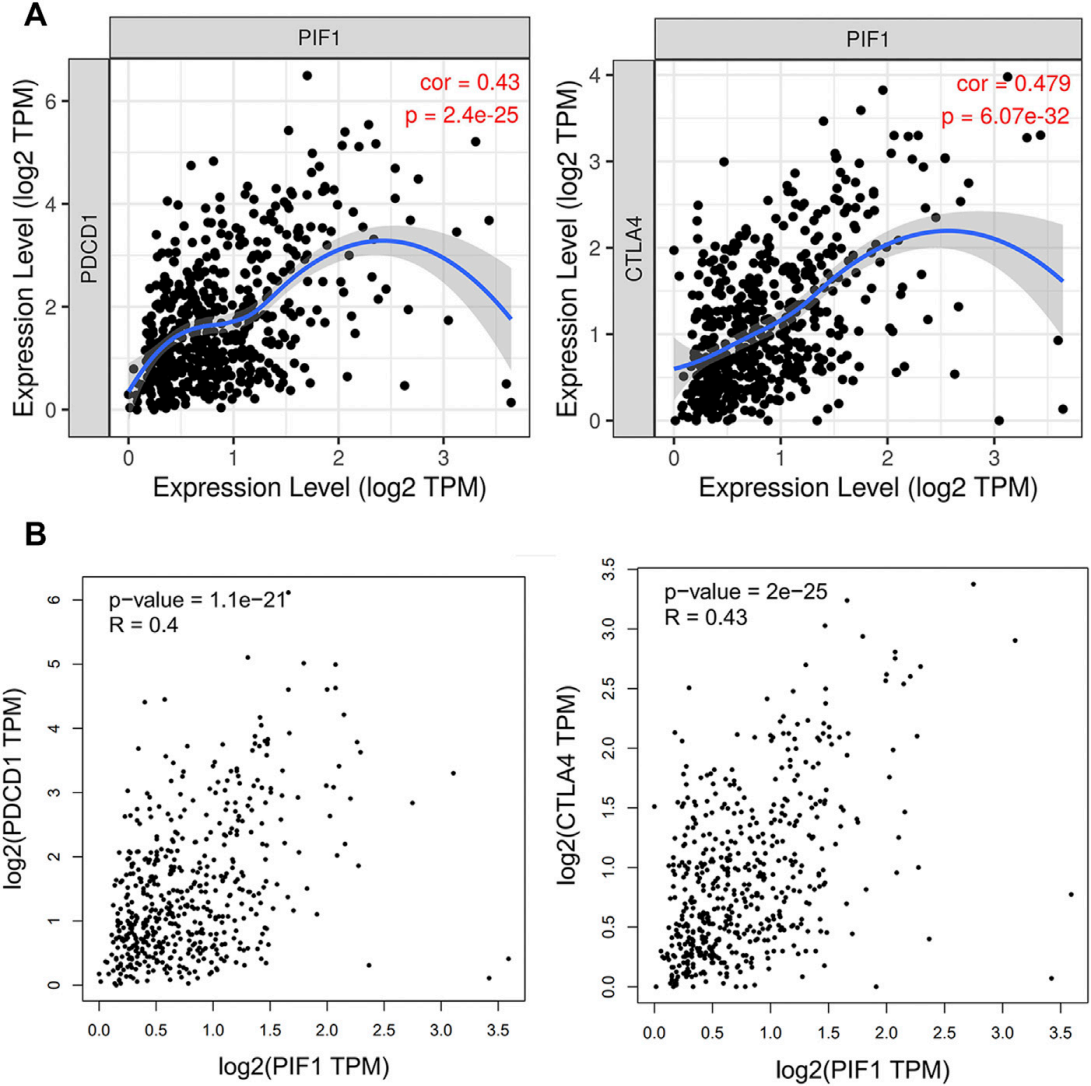
Relationship between PIF1 and PD1 in ccRCC. **(A)** Spearman correlation of PIF1 with the expression of PD-1/CTLA4 in ccRCC adjusted by purity using TIMER. **(B)** The expression correlation of PIF1 with PD-1/CTLA4 in ccRCC was determined by the GEPIA database. ccRCC: Clear Cell Renal Cell Carcinoma.

Due to the potential carcinogenic role of PIF1 in ccRCC, we evaluated the relationship of PIF1 with PD1/PD-L1 or CTLA4. As shown in Figures 9A,B, the expression of PIF1 was positively correlated with the expression of PD1 and CTLA4 in ccRCC. These results indicated that tumor immune escape might be involved in PIF1-mediated carcinogenesis of ccRCC.

## Discussion

In this study, we observed that the mRNA expression levels of PIF1 in different types of tumors were significantly different. Meanwhile, we found that both the mRNA and protein expression of PIF1 were upregulated in ccRCC. Then, we found that the upregulated mRNA expression of PIF1 is correlated with high TNM stage and high pathologic stage and proved that PIF1 can be an important reason for poor prognosis in patients with ccRCC. The ROC curve analysis showed that PIF1 could be a reference biomarker to effectively distinguish ccRCC from normal tissues. Based on the Kaplan‒Meier curves and univariate analysis, the high mRNA expression of PIF1 is related to short OS, DSS and PFI. In summary, the high mRNA expression of PIF1 could be a reference biomarker of worse prognosis in ccRCC. The nomogram provides a useful quantitative model for clinicians to help determine the correct prognosis of patients with ccRCC. Moreover, we constructed PPI networks and functional annotations.

PIF1 is a single gene encoded by the human genome, and it could have important implications for different solid tumors. In this research, we found that the expression of PIFI was significantly higher in urinary system diseases, such as ccRCC, bladder urothelial carcinoma (BLCA), cervical squamous cell carcinoma and endocervical adenocarcinoma (CESC), kidney renal clear cell carcinoma (KIRC), and testicular germ cell tumors (TGCTs). Furthermore, PIF1 expression was upregulated in other cancers of various organs, including stomach adenocarcinoma (STAD), sarcoma (SARC), pancreatic adenocarcinoma (PAAD), and others. Conclusion PIF1 may play an important role in the occurrence of tumors. Finally, this study also explored the relationship between PIF1 and PD1/CTLA4. The expression of PIF1 was positively correlated with PD1 and CTLA4 in ccRCC. This finding indicates that PIF1-mediated carcinogenesis may participate in the process of tumor immune escape in ccRCC.

Pif1 is a DNA helicase of SF1B, which exists extensively in the nucleus from yeast to humans, and it can unwind a forked DNA duplex repetitively. (Li et al., 2016). Research shows that the highly conserved PIF1 protein can maintain genomic stability not only by adjusting Okazaki fragment maturation (Budd et al., 2006), telomere homeostasis (Schulz and Zakian, 1994) and G-quadruplex DNA (G4-DNA) resolution (Ribeyre et al., 2009) but also through replication through DNA‒protein barriers (Sabouri et al., 2012) and DNA double-strand breaks (DBSs). (Wilson et al., 2013). Recently, several studies have pointed to the significant function of PIF1 in several different cancers, such as lung cancer, cervical cancer and neuroblastoma. However, the expression of PIF1 and its significance in diagnosis as a ccRCC biomarker have rarely been researched. In this study, we confirmed the differential expression of PIF1 in different types of store cancers based on pancancer analysis. Meanwhile, we also found that the expression of PIF1 in ccRCC was upregulated.

To date, the functions of PIF1 in cancer have not been studied and reported systematically. Previous studies suggested that the knockdown of PIF1 inhibited the growth of human NSCLC cells and promoted cell apoptosis and could serve as a potential therapeutic target for treating lung cancer. (Yin et al., 2021). Meanwhile, the lower expression of PIF1 can cause cell cycle arrest in the late G (1)/early S-phase and apoptosis in human breast cancer cell lines. (Chisholm et al., 2012). The expression of PIF1 can promote cervical cancer cell proliferation and inhibit cell apoptosis by upregulating the expression of telomerase TERT and increasing the rate of the G2/M phase. (Wang et al., 2020). In this study, the results showed that the expression of PIF1 was significantly related to organelle fission, nuclear division and mitotic nuclear division. Of course, more experiments are needed to prove the result. All these results demonstrated that PIF1 might be a potential molecular biomarker for cancer diagnosis or a novel target for cancer therapy. We used ROC curve analysis to prove the clinical value of PIF1 in the diagnosis of ccRCC. In our study, the results of the Kaplan‒Meier curves and log-rank test demonstrated that, compared to patients with low levels of PIF1, the decline in OS, DSS and PFI of ccRCC patients was associated with a high level of PIF1 mRNA expression. Meanwhile, PIF1 had a significantly greater AUC value in the identification of ccRCC. Based on these results, PIF1 can be used as a useful diagnostic marker of ccRCC and a helpful potential target for ccRCC treatment.

Meanwhile, we also found that the expression of PIF1 may have a potential and close relationship with immune cell infiltration. On the one hand, the study found that the expression of PIF1 was positively correlated with the expression of regulatory Treg cells and Th1 cells. It can inhibit the function of other immune cells and inhibit the antitumour immune response through various approaches, further tilting the antitumour response toward non-responsiveness. In addition, the expression of PIF1 was negatively correlated with Tgd, mast cells and neutrophils. They can exert an antitumour effect and increase the immune response. Previous research has shown that neutrophils are closely related to the prognosis of different tumors. (Donskov, 2013). Therefore, PIF1 overexpression in ccRCC may curb tumor immune responses involved in immune escape, further promoting cancer growth. Further research is needed to prove this relationship.

PIF1 is a highly conserved helicase that can maintain genomic stability. To date, its mechanism of action is not completely understood in tumor cells. Research shows that PIF1 plays a crucial role in the survival of human tumor cells, especially during replication stress, but this effect is not obvious in non-tumor cells and Pif1-deficient mice. The deletion of PIF1 can inhibit entry into S-phase upon release from thymidine-induced replication arrest and delay the progression of S-phase in human tumor cells. (Donskov, 2013). *In vitro* experiments show that purified full-length PIF1 can unwind and bind DNA structures that might form during DNA replication stress, containing G-quadruplexes (Sanders, 2010) as well as structures resembling stalled replication forks. (Sanders, 2010). Under normal cycling conditions and after ligand-induced G4 structure stabilization, the loss of PIF1 may lead to increased fork stalling as well as reduced replication fork rates. The folding of G4-structures, which were located at ssDNA GC-rich regions of lagging as well as leading strands, can damage fork movement during replication, which affects the stability of gene expression. (Mirkin and Mirkin, 2007; Mirkin, 2013; Tarsounas and Tijsterman, 2013). Research shows that G4-structure formation is influenced by the replication stress increase during the overexpression of the oncogene, which can increase the PIF1-dependence of fork progression. (Di Micco et al., 2006). Notwithstanding, whether the influence of PIF1 deletion on DNA replication progression only occurred to G4-regions of the human genome still needs to be further improved and validated. According to recent studies, the consensus G4 motif, which can form the G4 structure, is related to replication origin selection, replication efficiency and timing in human cells. (Besnard et al., 2012). PIF1 can change origin-firing processes by resolving the G4 structures. (Tarsounas and Tijsterman, 2013). The decreased percentage of striking similarities between the fork rate of non-telomeric regions (35.13%) and the new S-phase entry (38.49%) in parental fibroblasts with PIF1 depletion. (Tarsounas and Tijsterman, 2013). In vivo studies, PIF1 was related to the essential replication initiation cofactor CDC45 (Boos et al., 2012), and the interaction of endogenous CDC45 and C-terminal-FLAG-tagged PIF1 in human tumor cells was confirmed by coimmunoprecipitation. (Yataro et al., 2012). Although we have found that the expression of p21 proteins is increased with PIF1 depletion in HCT116 cells, the indirect effects of PIF1 on the resumption of S-phase entry and DNA replication cannot be ruled out. (Gagou et al., 2011). Therefore, the effect of PIF1 on the firing of S-phase entry and replication origins still needs further study.

There are a few notable limitations in the present study. First, the expression and prognostic significance of PIF1 were examined in publicly available online databases, and further research also needs to be validated in clinical samples to confirm the above results. Second, to study the precise impact of PIF1 on immune infiltration in ccRCC, further *in vivo*/*in vitro* experiments are required to confirm this result.

## Conclusion

In summary, in the present research, we report here for the first time that the mRNA and protein expression of PIF1 is upregulated in ccRCC and positively correlated with high TNM stage. This research demonstrates that PIF1 could be used as a reference biomarker to identify ccRCC patients with poor prognosis. PIF1 may play a distinct role in the microenvironment of ccRCC by regulating the tumor infiltration of immune cells, which is a new therapeutic target to affect the growth of the tumor.

## Data Availability

The datasets presented in this study can be found in online repositories. The names of the repository/repositories and accession number(s) can be found in the article/Supplementary Material.
