# Supplementary material for: Up-regulated PIF1 predicts poor clinical outcomes and correlates with low immune infiltrates in clear cell renal cell carcinoma
Source: Front Genet. 2023 Jan 4;13:1058040. doi: 10.3389/fgene.2022.1058040 (PMC9847676; doi:10.3389/fgene.2022.1058040)
Supplement: Supplementary file 1 [file DataSheet1.ZIP › raw data/┤·┬δ1.docx]

差异分析：

library(DESeq2)

(colData <- data.frame(row.names=colnames(t(rankTumorexprset)), #colData就样本分组list

group_list=samplelist) )#samplelist就是样本分组信息

dds <- DESeqDataSetFromMatrix(countData = rankTumorexprset,#表达谱文件，注意矩阵的方向和samplelist相反，并且要na.omit

colData = colData,

design = ~ expression)#expression是样本分组的列名

dds <- DESeq(dds)#这一步骤是最最长的

save(dds,file = 'DESeq2-dds.Rdata')

res <- results(dds,

contrast=c("expression","low","high"))

resOrdered <- res[order(res$padj),]

head(resOrdered)

DEG =as.data.frame(resOrdered)

DESeq2_DEG = na.omit(DEG)

nrDEG=DESeq2_DEG[,c(2,6)]

colnames(nrDEG)=c('log2FoldChange','pvalue')

火山图

library("ggplot2")

p<-ggplot(temp,aes(x=temp$logFC,y=-log10(temp$p.value)))+xlab("log2 Fold Change")+ylab("-log10P-Value")+

geom_point(size=4,alpha=0.6)

p

temp$threshold[temp$p.value < 0.05 & temp$logFC>0 ] = "up"

temp$threshold[temp$p.value < 0.05 & temp$logFC<0 ] = "down"

temp$threshold[temp$p.value > 0.05 & (temp$logFC>=0 | temp$logFC <= 0)] = "non"

p<-ggplot(temp,aes(x=temp$logFC,y=-log10(temp$p.value),colour=threshold))+xlab("log2 Fold Change")+ylab("-log10P-Value")+

geom_point(size=4,alpha=0.6)+

scale_color_manual(values =c("#0072B5","grey","#BC3C28")) #设置点的颜色

p

箱线图

## 安装R包

#install.packages("ggpubr")

## 加载R包

library("ggpubr")

## 导入数据

traits <- read.csv("data.csv",header=T,row.names = 1)

## 绘制箱线图

colnames(traits)

p <- ggboxplot(traits, x="sample", y="data",color="sample",palette=c("blue","#00AFBB","#E7B800","#FC4E07"),add="jitter",shape="sample")

p

## 输入进行差异显著性检验的组4组

groups <- list(c("g1","g2"))

## 在图中加入差异显著性检验结果

p + stat_compare_means(comparisons=groups,method="t.test",label="p.signif")

## 在图中加入差异显著性检验结果

p + stat_compare_means(comparisons=groups,method="t.test",label="p.signif")

列线图

install.packages('rms')

rm(list = ls())

#zairu

library(survival)

library(rms)

mydata<-read.csv("data2.csv",row.names=1,header=T)

colnames(mydata)

mydata<-mydata[c(1:2,4:10,12:16)]

mydata<-as.data.frame(mydata)

head(mydata)

#打包数据

attach(mydata)

dd<-datadist(mydata)

options(datadist='dd')

#拟合模型，并展示模型拟合的结果与模型参数。注意：可直接读取模型中Rank Discrim.参数 C，即为fit1模型的C-statistics。

fit1<-lrm(ICU~data1+ data2+ data3,data=mydata,x=T,y=T)

fit1

#构建列线图对象nom1, 打印列线图，结果如下图所示

nom <- nomogram(fit1, fun=plogis,fun.at=c(.001, .01, .05, seq(.1,.9, by=.1), .95, .99, .999),lp=F, funlabel="Low weight rate")

plot(nom)

#构建校准曲线对象cal1,打印校准曲线,结果如下。

cal1 <- calibrate(fit1, method='boot', B=1000)

plot(cal1,xlim=c(0,1.0),ylim=c(0,1.0))

#计算C指数

c<-rcorrcens(ICU~predict(fit1, newdata=mydata),data=mydata)

c[1,1]

c[1,1]-1.96*c[1,4]/2

c[1,1]+1.96*c[1,4]/2

##提取数值和公式

mydata$pred1 <- predict(fit1, mydata, type = "fitted")

mydata$linear.predictor <- predict(fit1, mydata, type = "lp")

write.csv(mydata,"mydata1.0.CSV")

ROC曲线

#install.packages("pROC")

#install.packages("ggplot2")

library(pROC) # 加载pROC包

library(ggplot2) # 调用ggplot2包以利用ggroc函数

aSAH<-read.csv("mydata1.0.CSV",row.names=1,header=T)

roc1 <- roc(aSAH$ICU,aSAH$HB);roc1 # Build a ROC object and compute the AUC

roc2 <- roc(aSAH$ICU, aSAH$Respiratory.failure);roc2

roc3 <- roc(aSAH$ICU, aSAH$APACHE);roc3# Create a few more curves for the next examples

roc4 <- roc(aSAH$ICU, aSAH$linear.predictor);roc4

# smooth=TRUE 绘制光滑曲线，默认为F，可省略

plot(roc1,

print.auc=TRUE, print.auc.x=0.4, print.auc.y=0.5,

# 图像上输出AUC值,坐标为（x，y）

auc.polygon=TRUE, auc.polygon.col="#fff7f7", # 设置ROC曲线下填充色

max.auc.polygon=FALSE, # 填充整个图像

grid=c(0.5, 0.2), grid.col=c("black", "black"), # 设置间距为0.1，0.2，线条颜色

print.thres=TRUE, print.thres.cex=0.9, # 图像上输出最佳截断值，字体缩放倍数

smooth=T, # 绘制不平滑曲线

main="Comparison of two ROC curves", # 添加标题

col="#FF2E63", # 曲线颜色

legacy.axes=TRUE) # 使横轴从0到1，表示为1-特异度

plot.roc(roc2,

add=T, # 增加曲线

col="#252A34", # 曲线颜色为红色

print.thres=TRUE, print.thres.cex=0.9, # 图像上输出最佳截断值，字体缩放倍数

print.auc=TRUE, print.auc.x=0.4,print.auc.y=0.4,

# 图像上输出AUC值,坐标为（x，y）

smooth = F) # 绘制不平滑曲线

plot.roc(roc3,

add=T, # 增加曲线

col="green", # 曲线颜色为红色

print.thres=TRUE, print.thres.cex=0.9, # 图像上输出最佳截断值，字体缩放倍数

print.auc=TRUE, print.auc.x=0.4,print.auc.y=0.3,

# 图像上输出AUC值,坐标为（x，y）

smooth = F) # 绘制不平滑曲线

plot.roc(roc4,

add=T, # 增加曲线

col="#FF2400FF", # 曲线颜色为红色

print.thres=TRUE, print.thres.cex=0.9, # 图像上输出最佳截断值，字体缩放倍数

print.auc=TRUE, print.auc.x=0.4,print.auc.y=0.2,

# 图像上输出AUC值,坐标为（x，y）

smooth = F) # 绘制不平滑曲线

部分统计通过在线软件完成。
